# Palliative Radiotherapy for Dysphagia in Esophageal Cancer: Short-Term Benefits and Late Toxicity Risks

**DOI:** 10.7759/cureus.94605

**Published:** 2025-10-14

**Authors:** Shingo Hashimoto, Yutaro Koide, Hiroyuki Tachibana, Masamune Noguchi, Yurika Shindo, Takahiro Aoyama, Shigenori Kadowaki, Masahiro Tajika, Tetsuya Abe, Takeshi Kodaira

**Affiliations:** 1 Radiation Oncology, Aichi Cancer Center, Nagoya, JPN; 2 Radiation Oncology, Japanese Red Cross Aichi Medical Center Nagoya Daiichi Hospital, Nagoya, JPN; 3 Clinical Oncology, Aichi Cancer Center, Nagoya, JPN; 4 Endoscopy, Aichi Cancer Center, Nagoya, JPN; 5 Gastroenterological Surgery, Aichi Cancer Center, Nagoya, JPN

**Keywords:** adverse events, dysphagia, esophageal cancer, palliative radiotherapy, quality of life, symptom relief

## Abstract

Introduction

Esophageal cancer is often diagnosed at an advanced stage, with dysphagia being the most common and distressing symptom that significantly impairs quality of life (QOL). Palliative radiotherapy is frequently used to relieve dysphagia, but its benefits and risks remain unclear. This study aimed to evaluate short-term dysphagia improvement and treatment-related toxicities in patients with esophageal cancer receiving palliative radiotherapy.

Materials and methods

We retrospectively analyzed 58 patients with esophageal cancer and dysphagia treated with palliative radiotherapy (30-40 Gy in 10-20 fractions) between April 2016 and March 2024. Of these, four patients (7%) received radiotherapy alone, while the remaining 54 (93%) underwent concurrent chemotherapy. Dysphagia scores were assessed before radiotherapy and one month after. Overall survival (OS) and treatment-related toxicities were also evaluated.

Results

Following radiotherapy, 45 of 58 patients (78%) experienced improvement in dysphagia, with a median time to symptom relief of 35 days. Among these 45 patients, recurrent dysphagia occurred in 17 (38%), with a median time from improvement to recurrence of 4.3 months (interquartile range (IQR), 2.2 to 6.0 months) and a median survival of 1.2 months after recurrence (IQR, 0.6 to 2.0 months). Across all patients, those who received concurrent chemotherapy tended to have higher rates of dysphagia improvement, suggesting that combination treatment may further alleviate esophageal obstruction. In patients with T3 disease or earlier, palliative radiotherapy was generally safe, and symptom relief contributed to improved oral intake and potential QOL benefits. In contrast, patients with T4b disease had a high incidence of late grade 2 to 3 esophageal or bronchial fistulas (36%), significantly more frequent than in T3 cases (6.7%). Acute toxicities were generally mild, but the risk of serious late adverse events in advanced disease highlights the need for careful patient selection.

Conclusion

Palliative radiotherapy can improve short-term dysphagia in most patients with esophageal cancer; however, patients with T4b disease face a higher risk of serious late adverse events, necessitating careful selection. While symptom relief may temporarily improve QOL, clinicians should weigh these risks carefully and discuss them with patients before proceeding. Patients should be counseled not only about the potential for short-term symptom relief but also about the risks of dysphagia recurrence and serious late adverse events to support shared decision-making.

## Introduction

Esophageal cancer ranks sixth in cancer deaths and eighth in global incidence [1]. More than half of patients are diagnosed at an advanced stage or with distant metastases, often presenting with tumor-related symptoms and general health deterioration [2]. Dysphagia is the most common and distressing complication, worsening with disease progression and leading to malnutrition, aspiration pneumonia, pain, and decreased performance status (PS) [3,4]. Therefore, sustained relief of dysphagia is a critical goal to improve quality of life (QOL) in patients with advanced or refractory esophageal cancer.

Treatment options for malignant dysphagia include external beam radiotherapy (EBRT), endoluminal brachytherapy, metallic stenting, balloon dilation, bypass surgery, and chemotherapy [5,6]. Although esophageal stents provide rapid symptom relief, they carry a high risk of severe adverse events, such as perforation, bleeding, and fistula formation. Chemotherapy and chemoradiotherapy (CRT) can improve swallowing function and nutritional status but often involve systemic toxicities.

Palliative radiotherapy is generally indicated for patients with advanced esophageal cancer who are unsuitable for curative treatment or who require symptom control, particularly for dysphagia, pain, or bleeding [7,8]. Common acute adverse events include esophagitis, nausea, and fatigue, whereas late complications can include esophageal stricture, ulceration, and rarely, fistula formation [9-11]. Despite these risks, palliative radiotherapy can provide meaningful symptomatic relief with a relatively favorable safety profile, especially when carefully planned and delivered [7-11]. Accordingly, the present study aimed to evaluate the short-term efficacy and treatment-related toxicities of palliative radiotherapy in patients with esophageal cancer and dysphagia, with a focus on balancing symptomatic benefit against the risk of serious late adverse events.

## Materials and methods

This study was approved by the Institutional Review Board of Aichi Cancer Center (approval no. 2024-0-354) and was performed in line with the principles of the Declaration of Helsinki. We retrospectively analyzed 58 advanced esophageal cancer patients who underwent palliative radiotherapy for dysphagia at our hospital from April 2016 to March 2024. Dysphagia was assessed using the Ogilvie dysphagia scale (Table 1) [12].

**Table 1:** Dysphagia scoring scale Based on the Ogilvie dysphagia scale [12]. This scale is not copyrighted and is freely available for academic use.

Score	Description
0	Able to consume a normal diet
1	Dysphagia with certain solid foods
2	Able to swallow semi-solid soft foods
3	Able to swallow liquids only
4	Unable to swallow saliva (complete dysphagia)

Inclusion and exclusion criteria

Patients with (1) histologically confirmed esophageal cancer; (2) dysphagia score 1-4; (3) stage Ⅲ or Ⅳ disease deemed unsuitable for curative treatment by a multidisciplinary tumor board; and (4) who underwent radiotherapy delivered with a total dose of less than 50 Gy, which is below the threshold for curative intent in radiation oncology, were included in the study. The main reasons for exclusion from curative treatment were disease progression and distant metastases. Additionally, some patients received palliative radiotherapy as their only treatment option due to poor PS or advanced age.

Pretreatment evaluation included history, physical exam, blood tests, CT of the neck/chest/abdomen, esophagogastroscopy, and endoscopy. Bronchoscopy was performed when clinically indicated. The disease stage was diagnosed according to the 8th edition of the tumor, nodes, metastasis (TNM) staging classification of the Union for International Cancer Control [13]. Performance status was assessed by Eastern Cooperative Oncology Group criteria. 

Treatment

The EBRT used 6 or 10 MV X-rays from a linear accelerator, delivered five days per week. Chemotherapy was added when feasible; however, radiotherapy alone was administered to patients with poor PS or organ dysfunction. Treatment plans were decided by a multidisciplinary cancer board including gastroenterology, surgery, medical oncology, and radiation oncology specialists. The clinical target volume covered the primary tumor and nearby lymph node metastases affecting QOL; distant metastases were excluded. The planning target volume extended the clinical target by 2 cm vertically and 0.5 cm laterally for the primary tumor and by 0.5 cm around lymph nodes. Three-dimensional conformal radiotherapy with 2-4 fields was used.

Follow‑up and assessment

Patients were evaluated for dysphagia symptoms and radiotherapy-related toxicities during routine medical examinations. Toxicities, excluding dysphagia, were graded based on the Common Terminology Criteria for Adverse Events (CTCAE), version 5.0 [14]. Dysphagia scores (Table 1) were recorded at baseline (before radiotherapy) and one month after radiotherapy. Toxicities occurring within three months were classified as acute; those after three months as late.

Statistical analysis

Overall survival (OS) was defined as the time from the start of radiotherapy to the last follow-up or death. The OS was estimated using the Kaplan-Meier method and compared using the log-rank test. Changes in dysphagia scores from baseline to one month post-radiotherapy were analyzed using the Wilcoxon signed-rank test, and subgroup comparisons (e.g., by radiation dose or T classification) were performed using the Mann-Whitney U test or Fisher’s exact test as appropriate. All statistical analyses were conducted using EZR version 1.68 (Saitama Medical Center, Jichi Medical University, Saitama, JPN) [15], with p < 0.05 considered significant. For univariate analysis, patients who received chemotherapy concurrently with radiotherapy were classified as the 'concurrent chemotherapy' group, whereas those who received chemotherapy only pre- or post-radiotherapy were included in the 'no concurrent chemotherapy' group. This classification was adopted to specifically evaluate the potential impact of concurrent chemotherapy on short-term dysphagia improvement.

## Results

Patient and tumor characteristics

Table 2 summarizes the patient and tumor characteristics, dose fractionation, and chemotherapy details. Patients with dysphagia from recurrent laryngeal nerve paralysis were not included. Chemotherapy mainly involved 5-fluorouracil with cisplatin or folinic acid, fluorouracil, and oxaliplatin (FOLFOX). Regarding the timing of chemotherapy relative to radiotherapy, 18 patients received chemotherapy prior to radiotherapy, 44 received concurrent chemotherapy, 45 received chemotherapy after radiotherapy, and four patients underwent palliative radiotherapy alone. Among these four patients, three experienced improvement in their subjective symptoms. Median follow-up was 7.1 months (range: 1.9-39). All patients completed their planned course of radiotherapy or chemoradiotherapy.

**Table 2:** Patient and tumor characteristics UICC 8th: Union for International Cancer Control 8th edition [13], Ce: Cervical esophagus, Ut: Upper thoracic esophagus, Mt: Middle thoracic esophagus, Lt: Lower thoracic esophagus, Ae: Abdominal esophagus

Characteristics	Category	Patients, total n = 58
Age (years)	mean (range), y	65 (36-90)
Gender	Male/female	48/10
Performance status	0/1 /2	11/41 /6
Primary tumor site	Ce/Ut/Mt/Lt/Ae	3/8/21/17/9
T stage	T1/T2/T3/T4a/T4b	0/2/45/0/11
N stage	N0/N1/N2/N3	1/13/23/21
M stage	M0/M1	10/48
UICC 8th stage	Ⅲ/ⅣA/ⅣB	4/6/48
Tumor length (cm)	Mean (range)	7.2 (3.0-17)
Histopathology	Squamous cell carcinoma	48
	Adenocarcinoma	5
	Neuroendocrine carcinoma	1
	Small cell carcinoma	3
	Carcinoma	1
Radiation dose	30 Gy in 10 fractions	10
	36 Gy in 12 fractions	2
	40 Gy in 20 fractions	46
Chemotherapy prior to radiotherapy	Yes/No	18/40
Concurrent chemotherapy	Yes/No	44/14
Chemotherapy after radiotherapy	Yes/No	45/13
Palliative radiotherapy alone	N/A	4

Evaluation of dysphagia and survival

**Figure 1:**
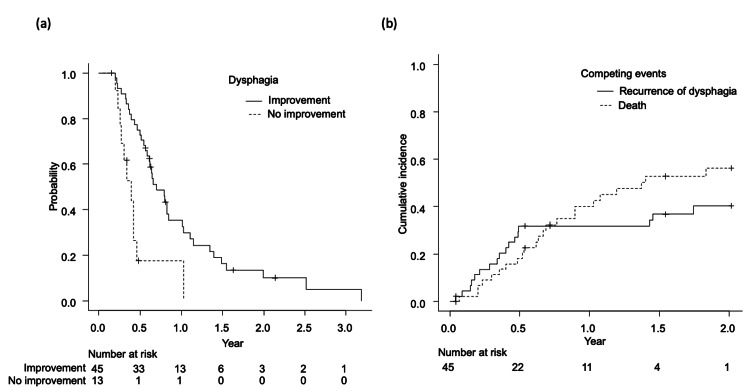
Analysis of outcomes (a) Overall survival comparison between patients whose dysphagia improved after treatment (solid line) and those whose dysphagia did not improve (dotted line) (total n = 58); (b) Cumulative incidence of recurrent dysphagia (solid line) and death (dotted line) in patients with improved dysphagia (n = 45)

Following treatment, dysphagia improved in 45 of 58 patients (78%). The median time from the initiation of radiotherapy to symptom improvement was 35 days. Patients with improved dysphagia had significantly longer OS than those without improvement (p < 0.05, Figure 1(a)). At six months, OS was 75% (95% CI: 60-85) in the improved group versus 18% (95% CI: 2.8-43) in the no improvement group. As shown in Figure 1(b), among the 45 patients who initially responded, 17 (38%) experienced recurrent dysphagia. The median time from improvement to recurrence was 4.3 months (interquartile range (IQR), 2.2-6.0 months), and the median time from recurrence to death was 1.2 months (IQR, 0.6-2.0 months).

A total of 58 patients with esophageal cancer who underwent palliative radiotherapy were analyzed for improvement in dysphagia. In univariate analysis using Fisher's exact test, there were no statistically significant differences in symptom improvement according to tumor type (squamous cell carcinoma (SCC) vs non-SCC, 75.0% vs 90.0%; p = 0.429), T stage (T4 vs T3 or less, 81.8% vs 76.6%; p = 1.000), stenosis length (< 7 cm vs ≥ 7 cm, 83.3% vs 73.5%; p = 0.526), or radiation dose (< 40 Gy vs ≥ 40 Gy, 58.3% vs 82.6%; p = 0.116). In contrast, concurrent chemotherapy was significantly associated with higher rates of dysphagia improvement (concurrent chemotherapy vs none, 86.4% vs 50.0%; p < 0.05). Overall, palliative radiotherapy without concurrent chemotherapy achieved symptomatic relief in many patients, but the addition of concurrent chemotherapy further enhanced the benefit (Table 3).

**Table 3:** Univariate analysis of factors associated with dysphagia improvement after palliative radiotherapy in esophageal cancer patients (total n = 58, Fisher’s exact test) Improvement rate = Improvement ÷ Total × 100, SCC: Squamous cell carcinoma

Factor	Category	Improvement	No improvement	Improvement rate (%)	p-value (Fisher)
Histology	Non-SCC	9	1	90	0.43
	SCC	36	12	75	
T stage	T3 or less	36	11	77	1.0
	T4	9	2	82	
Stenosis length	≥ 7 cm	25	9	74	0.53
	< 7 cm	20	4	83	
Radiation dose	≥ 40 Gy	38	8	83	0.12
	< 40 Gy	7	5	58	
Concurrent chemotherapy	Yes	38	6	86	< 0.05
	No	7	7	50	

**Figure 2:**
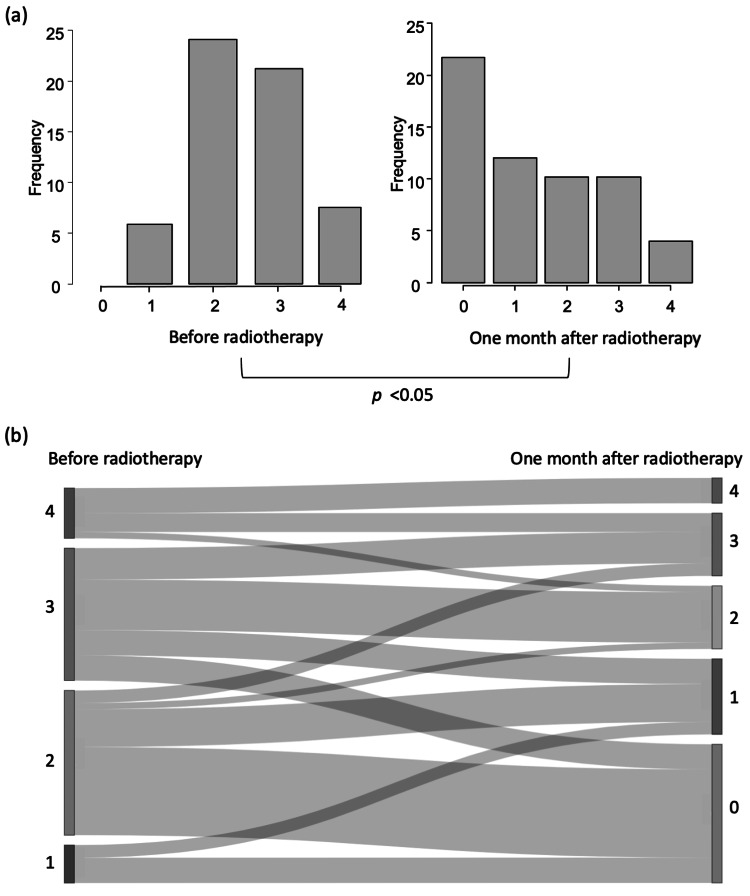
Analysis of dysphagia scores (total n = 58) (a) Changes in dysphagia scores (Wilcoxon signed-rank test); (b) Sankey diagram of dysphagia score transitions

**Figure 3:**
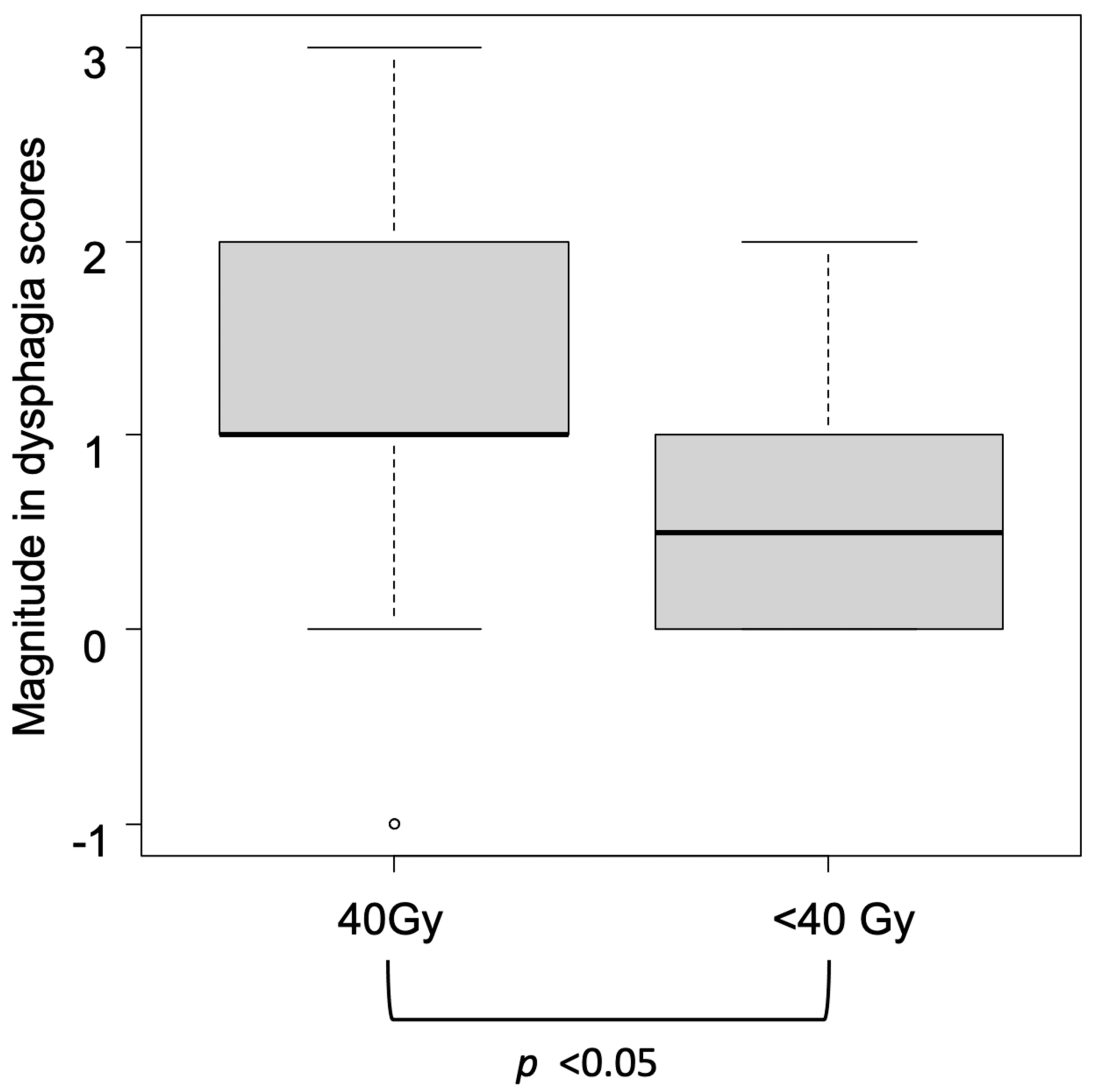
Magnitude of dysphagia score improvement from baseline to one month after radiotherapy (total n = 58, Mann–Whitney U test) The small circle indicates outliers.

Changes in dysphagia scores are shown in Figure 2. The Wilcoxon signed-rank test confirmed significant improvement from baseline to one month after radiotherapy (p < 0.05, Figure 2(a)). The Sankey diagram (Figure 2(b)) illustrates individual transitions in dysphagia grades. The magnitude of improvement in dysphagia scores from baseline to one month was significantly greater in patients who received 40 Gy than in those who received < 40 Gy, as assessed by the Mann-Whitney U test (p < 0.05, Figure 3).

Toxicity

Acute and late toxicities are summarized in Table 4. Among acute toxicities, grade 3 nausea occurred in one patient (1.7%); grade 2 and grade 3 esophagitis in three (5.2%) and one (1.7%) patients, respectively; and grade 3 febrile neutropenia in two patients (3.4%). Among late toxicities, grade 2 and grade 3 esophageal or bronchial fistulas were observed in five (8.6%) and two (3.4%) patients, respectively. Late grade ≥2 fistulas were observed in three of 45 patients with T3 disease (6.7%) and in four of 11 patients with T4b disease (36%), with a significantly higher incidence in the T4b group (Fisher’s exact test, p < 0.05). No such fistulas were observed in the two patients with T2 disease. No grade 4 or 5 toxicities were observed.

**Table 4:** Treatment toxicities (total n= 58) CTCAE: Common terminology criteria for adverse events [14]

CTCAE version 5.0			Grade 2	Grade 3
			n (%)	n (%)
Acute toxicities	Nausea		N/A	1 (1.7%)
	Esophagitis		3 (5.2%)	1 (1.7%)
	Febrile neutropenia		N/A	2 (3.4%)
Late toxicities	Esophageal or bronchial fistula		5 (8.6%)	2 (3.4%)

## Discussion

This study retrospectively evaluated short-term dysphagia improvement and treatment-related toxicities in esophageal cancer patients who received palliative radiotherapy. Based on assessments before and one month after radiotherapy, 78% of patients experienced improvement in dysphagia, with a median time to symptom relief of 35 days. Among these 45 patients, recurrent dysphagia occurred in 17 (38%), with a median time from improvement to recurrence of 4.3 months (IQR, 2.2-6.0 months) and a median survival of 1.2 months after recurrence (IQR, 0.6-2.0 months). Notably, symptomatic relief from dysphagia can be temporary, and recurrence may occur relatively early (as early as 2.2 months in the first quartile) after initial improvement, highlighting a limitation of palliative radiotherapy that should be discussed with patients. In patients with T3 disease or earlier, palliative radiotherapy was delivered relatively safely, and symptom relief was associated with potential improvements in quality of life (QOL). In many patients within the improvement group, better oral intake was achieved, contributing to greater independence in daily living and improved QOL. Notably, patients who received concurrent chemotherapy in addition to radiotherapy tended to show higher rates of dysphagia improvement, suggesting that combination treatment may further alleviate esophageal obstruction. Therefore, the observed effects should be interpreted as reflecting the additive effect of palliative radiotherapy in the context of chemotherapy-based treatment, rather than the effect of radiotherapy alone.

In contrast, among patients with T4b disease, the incidence of late-grade 2-3 esophageal or bronchial fistulas was high (27%), significantly more frequent than in T3 cases (8.9%). For this subgroup, the risks of QOL deterioration due to adverse events may outweigh potential benefits of symptom relief, underscoring the necessity of careful patient selection. Moreover, it remains difficult to isolate the therapeutic effects of radiotherapy alone, given the confounding influence of concurrent chemotherapy.

Previous studies have suggested that combined chemotherapy and local therapy may improve dysphagia and nutritional status in stage IVB esophageal cancer. Ikeda et al. [16] reported that 85% of patients were able to discontinue nutritional support after chemoradiotherapy (5-FU plus cisplatin), with a median OS of 8.3 months. In JCOG0303 [17], 22% of patients with locally advanced T4 esophageal cancer treated with chemoradiotherapy (60 Gy in 30 fractions with cisplatin and fluorouracil) developed esophageal fistulas, indicating a high risk of severe toxicity.

Similarly, in a study of 70 patients with esophageal SCC, radiotherapy alone (30 Gy in 10 fractions) was compared with concurrent chemoradiotherapy (40 Gy in 20 fractions) [18]. Dysphagia improved in 60% of patients in both groups, but the chemoradiotherapy group achieved significantly longer nutritional support-free survival. Factors associated with dysphagia improvement included tumor length <6 cm, tumor circumference <3/4, and chemoradiotherapy with 40 Gy in 20 fractions. However, a multicenter trial using short-course radiotherapy (30-35 Gy in 10-15 fractions) showed no significant differences in dysphagia relief or overall survival between chemoradiotherapy and radiotherapy alone, despite slight improvements in the chemoradiotherapy group [19]. Moreover, chemoradiotherapy was associated with increased adverse events, including hematologic toxicity. These findings suggest that while chemoradiotherapy may provide some incremental benefit, short-course radiotherapy remains a reasonable option for patients with limited life expectancy, such as those with poor performance status or widespread metastases.

Esophageal stenting is another widely used option for malignant dysphagia. Although stents can provide rapid symptom relief, they are associated with a higher risk of severe adverse events, including fistulas, perforation, and bleeding [20]. In contrast, radiotherapy has fewer adverse effects, provides better pain relief, and achieves comparable dysphagia improvement [20-23]. The results of our study are consistent with these reports: in patients with T3 disease or earlier, palliative radiotherapy can be delivered safely, with meaningful symptom improvement and potential QOL benefits.

This study has several limitations. Dysphagia assessment was limited to two time points (before radiotherapy and one month after), and thus long-term outcomes such as recurrence of dysphagia or durability of symptom relief could not be adequately evaluated. In addition, most patients received chemotherapy, making it difficult to separate the effects of radiotherapy alone; therefore, the observed outcomes should be considered as the additive effect in the context of chemotherapy-based treatment. When proposing palliative radiotherapy, clinicians should provide a comprehensive overview of potential benefits and risks and engage patients in shared decision-making based on their values and preferences. Multivariate analyses were not feasible due to the limited sample size and small number of events, and therefore only univariate analyses were performed. Finally, this is a retrospective, single-institution analysis with heterogeneity in treatment regimens and radiation protocols, which limits the generalizability of the findings.

From a clinical perspective, palliative radiotherapy may improve short-term swallowing function and QOL in patients with T3 disease or earlier, but its use in T4b patients requires extreme caution due to the high risk of serious late adverse events. When deciding on treatment, factors such as tumor site, patient condition, expected survival, and the use of concurrent therapies should be carefully considered. Further prospective studies are needed to evaluate the safety and efficacy of radiotherapy, with or without chemotherapy or stenting, to optimize palliative treatment strategies in complex clinical settings.

## Conclusions

In this retrospective study, palliative radiotherapy provided a short-term improvement in dysphagia in the majority of esophageal cancer patients, particularly those with T3 disease or earlier. Across all patients, those who received concurrent chemotherapy tended to have higher rates of dysphagia improvement, suggesting that combination treatment may further alleviate esophageal obstruction. However, in patients with T4b disease, the risk of severe late adverse events, such as esophageal or bronchial fistulas, was substantial and may outweigh the potential benefits of treatment. Careful patient selection and individualized risk-benefit assessment are therefore essential when considering palliative radiotherapy for esophageal cancer.
